# Chilling and frost tolerance in *Miscanthus* and *Saccharum* genotypes bred for cool temperate climates

**DOI:** 10.1093/jxb/eru105

**Published:** 2014-03-18

**Authors:** Patrick C. Friesen, Murilo M. Peixoto, Florian A. Busch, Daniel C. Johnson, Rowan F. Sage

**Affiliations:** ^1^Department of Ecology and Evolutionary Biology, University of Toronto, 25 Willcocks Street, Toronto, Ontario, Canada, M5S 3B2; ^2^Plant Science Division, Research School of Biology, Australian National University, Canberra, ACT, 0200Australia; ^3^Department of Cell and Systems Biology, University of Toronto, 25 Harbord Street, Toronto, Ontario, Canada, M5S 3G5

**Keywords:** Chilling tolerance, chlorophyll fluorescence, gas exchange, *Miscanthus*, perennial C4 grasses, photosynthesis, *Saccharum*.

## Abstract

*Miscanthus* hybrids are leading candidates for bioenergy feedstocks in mid to high latitudes of North America and Eurasia, due to high productivity associated with the C_4_ photosynthetic pathway and their tolerance of cooler conditions. However, as C_4_ plants, they may lack tolerance of chilling conditions (0–10 °C) and frost, particularly when compared with candidate C_3_ crops at high latitudes. In higher latitudes, cold tolerance is particularly important if the feedstock is to utilize fully the long, early-season days of May and June. Here, leaf gas exchange and fluorescence are used to assess chilling tolerance of photosynthesis in five *Miscanthus* hybrids bred for cold tolerance, a complex *Saccharum* hybrid (energycane), and an upland sugarcane variety with some chilling tolerance. The chilling treatment consisted of transferring warm-grown plants (25/20 °C day/night growth temperatures) to chilling (12/5 °C) conditions for 1 week, followed by assessing recovery after return to warm temperatures. Chilling tolerance was also evaluated in outdoor, spring-grown *Miscanthus* genotypes before and after a cold front that was punctuated by a frost event. *Miscanthus×giganteus* was found to be the most chilling-tolerant genotype based on its ability to maintain a high net CO_2_ assimilation rate (*A*) during chilling, and recover *A* to a greater degree following a return to warm conditions. This was associated with increasing its capacity for short-term dark-reversible photoprotective processes (Φ_REG_) and the proportion of open photosystem II reaction centres (qL) while minimizing photoinactivation (Φ_NF_). Similarly, in the field, *M.×giganteus* exhibited a significantly greater *A* and pre-dawn *F*_v_/*F*_m_ after the cold front compared with the other chilling-sensitive *Miscanthus* hybrids.

## Summary

Triploid *Miscanthus* hybrids have superior chilling tolerance across *Miscanthus* and *Saccharum* genotypes bred for cool temperate climates.

## Introduction

Hybrids of the C_4_ grass species *Miscanthus sacchariflorus* and *M. sinensis* are leading candidates for second-generation bioenergy feedstocks of cool-temperate climates (Deuter, 2000; Heaton *et al.*, 2010; Zub and Brancourt-Hulmel, 2010; Jones, 2011). *Miscanthus* hybrids are more chilling tolerant than most C_4_ crops such as maize and sugarcane, and can be freezing tolerant in the winter dormant state (Clifton-Brown and Lewandowski, 2000; Naidu *et al.*, 2003; Farrell *et al.*, 2006; Wang *et al.*, 2008). Relative to other cultivated C_4_ species, *Miscanthus* hybrids exhibit higher net CO_2_ assimilation rates (*A*) at cool temperatures (10–18 **°**C), and produce a canopy with a leaf area index >1 well before other C_4_ crops of the temperate zone (Beale and Long, 1995; Beale *et al.*, 1996; Dohleman and Long, 2009; Dohleman *et al.*, 2009). This ability to produce an early-season canopy is critical for the success of *Miscanthus* varieties in higher latitudes, because it allows them to exploit the long photoperiods of May and June. However, production of an early-season canopy increases the risk of severe injury due to chilling temperatures (<12 °C) and episodic frost events that can be common in cool temperate to boreal climates.

C_4_ plants are generally vulnerable to chilling conditions, and most lack frost tolerance (Sage *et al.*, 2011; Long and Spence, 2013). Thus, a potential limitation in using C_4_ plants to exploit the abundant land and long summer days at higher latitudes is chilling sensitivity that may harm the C_4_ photosynthetic apparatus early in the growing season. In the case of *Miscanthus*, its tolerance of cool conditions has not been associated with frost tolerance or tolerance of chilling temperatures below 8–10 **°**C (Farage *et al.*, 1998, 2006). While better than most C_4_ species, *Miscanthus* may still suffer severe stress as it grows a new canopy early in the growing season. In C_4_ plants, this stress may induce severe photoinhibition, or damage enzymes of the C_4_ metabolic cycle, notably pyruvate orthophosphate dikinase (PPDK) and NADP-malate dehydrogenase (NADP-MDH) (Long, 1983; Potvin *et al.*, 1986; Du *et al.*, 1999*a*; Naidu *et al.*, 2003; Wang *et al.*, 2008). In addition, even if they express cold-tolerant isoforms of C_4_ cycle enzymes (as shown for *Echinochloa crus-galli*, for example; Simon and Hatch, 1994), cold-tolerant C_4_ species may be limited by Rubisco capacity at low temperatures (Kubien and Sage, 2004*b*; Sage *et al.*, 2011). In addition to placing a low ceiling on photosynthetic capacity, a Rubisco limitation may restrict photochemical quenching and predispose C_4_ species to photoinhibition in chilly conditions (Kubien *et al.*, 2003; Kubien and Sage, 2004*a*; Long and Spence, 2013).

To improve the cold tolerance of *Miscanthus*, novel hybrids have been bred from wild accessions of *M. sinensis* and *M. sacchariflorus* collected in colder areas of their distribution ranges in eastern Asia (Deuter, 2000; Heaton *et al.*, 2010). In addition, crosses between cold-adapted subtropical species of *Saccharum*, notably *S. spontaneum*, and commercial sugarcane (*Saccharum officinarum*) have generated new ‘energycane’ cultivars that promise to be tolerant of episodic chilling present in warm temperate climates (Khan *et al.*, 2013). Sugarcane is currently the most productive bioenergy feedstock on earth, but its growth is largely restricted to tropical and subtropical zones due to its chilling intolerance (Du *et al.*, 1999*a*, *b*). Sugarcane varieties with some chilling tolerance are grown at higher elevation at low latitudes; however, the extent of their chilling tolerance compared with *Miscanthus* is uncertain. New energycane varieties are more productive than commercial sugarcane in the US Southeast, and these may allow for a high biomass sugarcane-like feedstock to be grown in much of the Earth’s temperate zone (Hale *et al.*, 2013; Knoll *et al.*, 2013).

In this study, the low temperature (5–12 **°**C) tolerance of the photosynthetic apparatus of (i) five *Miscanthus* cultivars bred or selected for cold tolerance; (ii) a putative chilling-tolerant energycane hybrid; and (iii) and an upland sugarcane variety that may have some chilling tolerance was evaluated (Deuter, 2000; Hale *et al.*, 2013). Using plants grown in plant growth chambers, whole-leaf gas exchange and pulse-amplitude modulated (PAM) fluorescence were used to characterize the photosynthetic responses to chilling conditions, and then evaluate post-chilling recovery. Photosynthetic responses include net CO_2_ assimilation rate (*A*) and the response of *A* over a range of intercellular CO_2_ concentrations (*A*/*C*_i_ curves). Gas exchange was combined with fluorescence approaches that address mechanisms underlying photoinhibition leading to sustained non-photochemical quenching (NPQ). The approach was to partition photoprotective processes that relax after an hour of dark adaptation (Φ_REG_) from cumulative photoinactivation (Φ_NF_) as well as measure the proportion of open photosystem II (PSII) reaction centres (qL) under high light. The proportion of open PSII reaction centres (qL) reflects the redox state of the Q_A_ pool of plastoquinones; a greater qL indicates that the Q_A_ pool is more oxidized and probably has a greater capacity for linear electron transport. The results of a field study conducted in parallel with growth chamber experiments to characterize impacts of early-season chilling and frost on the same five *Miscanthus* genotypes plus a variety of *M. sinensis* whose leaves survived overnight frost are also reported.

## Materials and methods

### Plant material and cultivar selection

*Miscanthus* cultivars used in the growth chamber study were provided by New Energy Farms Ltd of Leamington Ontario, Canada (http://newenergyfarms.com/site/index.html) and were collected from their field site near Leamington. Energycane was supplied by New Energy Farms Ltd and was originally bred at the Sugarcane Research Unit of the USDA-ARS, in Houma, Louisiana, USA (Dean Tiessen of New Energy Farms, personal communication, November 2013). The variety of energycane is ‘Ho 02-113’, an F_1_ *Saccharum* hybrid of an *S. spontaneum* ecotype from the Himalayan foothills of northern India (SES 234) and a leading commercial sugarcane variety in LA, USA, a complex hybrid of *S. officinarum×S. spontaneum×S. barberi×S. sinense* (LCP 85–384) (Milligan *et al.*, 1994; Hale *et al.*, 2013). Hawaiian upland sugarcane (ULSC, *Saccharum officinarum* cv. H78-3567) is a commercial variety provided by Albert Arcinas (Hawaiian Agricultural Research Centre, Kunia, HI, USA). *Miscanthus×giganteus* (*M161*) was originally collected from the Chicago Botanical Gardens and has been the research standard at the University of Illinois at Urbana-Champaign since 1988 (Heaton *et al.*, 2010). The putative origin of *M.×giganteus* is southern Japan where tetraploid *M. sacchariflorus* and diploid *M. sinensis* overlap (Lewandowski *et al.*, 2000; Heaton *et al.*, 2010; Nishiwaki *et al.*, 2011). All other *Miscanthus* hybrids were bred by Dr Martin Deuter and Dr Juergen Abraham of Tinplant Biotechnik GmbH (http://www.tinplant-gmbh.de/, Klein-Wanzleben, Germany) and are crosses between *M. sacchariflorus* and *M. sinensis*. *Miscanthus* 116 (‘Nagara’) is an allotriploid with the maternal tetraploid *M. sacchariflorus* parent from around the Nagara River in Japan. *Miscanthus* 118 is an allotetraploid with the same maternal line as *M116*. *Miscanthus* 147 and *M115* (Amuri lines) are allodiploid and have different but closely related maternal *M. sacchariflorus* parents from the Amur River in north-east Asia (G. van Koeverden of New Energy Farms, Leamington, ON, Canada, personal communication, August 2010).

### Growth chamber experiment

Single rhizomes of each genotype were planted in 20 litre pots filled with a mixture of 40% triple mix (topsoil, sand, and compost blend), 40% coarse sand, and 20% ProMix (Premier Tech, Quebec, Canada). Plants were then grown in a controlled environment chamber (Enconair ‘Bigfoot’ series, Winnipeg, MB, Canada) at 25/20 **°**C (day/night leaf temperatures) under a 14h day/10h night cycle and rotated around the chamber daily to minimize within-chamber effects. Pots were insulated to prevent severe chilling of roots by the cold floor of the growth chamber, which was observed in preliminary trials. Growth light intensities were 550±50 μmol photons m^–2^ s^–1^ on the upper leaf canopy. Plants were watered daily and fertilized twice weekly with a mixture of 28-10-8 and 30-10-10 NPK commercial fertilizers (Miracle-Gro, The Scotts Company LLC) at the manufacturer’s suggested concentration. Fertilizer solutions were supplemented with 0.016M CaNO_3_ and 0.001M MgSO_4_ weekly. Plants were exposed to chilling treatments when 2–4 fully expanded leaves were present. After a day of warm measurements, chilling temperatures of 12/5 **°**C (day/night) were initiated beginning the morning after the lights came on. Chilling temperatures were continued for 6 d. Warm temperatures (25**/**20 **°**C) were re-established on the morning of the seventh day.

### Gas exchange and chlorophyll fluorescence

Leaf gas exchange and chlorophyll fluorescence were measured on a single youngest fully expanded leaf of a randomly selected shoot the day before chilling (day 0), days 1, 2, 4, and 6 during the chilling treatment, and the day after the return to warm temperatures (day 7). An open-path gas exchange system (LI-6400; Li-Cor, Lincoln, NE, USA) measured photosynthetic parameters. Leaf temperatures during the gas exchange measurements were 25 °C before chilling (when the growth temperature was 25/20 °C), 11 °C during chilling (12/5 °C growth conditions), and 25 °C upon return to warm growth temperatures (25/20 °C). Measurements were initially conducted at ambient CO_2_ conditions of 380 μmol mol^–1^, saturating light intensity (1800 μmol photons m^–2^ s^–1^), and a leaf to air vapour pressure difference of 1–3 kPa. After the light intensity was brought to saturation, the response of *A* to a range of intercellular CO_2_ concentrations (*C*_i_) was measured. Carboxylation efficiency (CE) was calculated as the initial slope of the *A*/*C*_i_ response, which is the linear portion that included all points below a *C*_i_ of 75 μmol mol^–1^ at 25 °C and *C*_i_ <40 μmol mol^–1^ at 11 °C.

Chlorophyll fluorescence was measured using a PAM-2100 pulse amplitude-modulated fluorometer and the PAM-2100 dark leaf clips to ensure uniform distance of the fluorescence probe from the leaf (Heinz Walz, Effeltrich, Germany; http://www.walz.com). Initially, leaves were exposed to ~1800 μmol photons m^–2^ s^–1^ of actinic light for 5min to measure the steady-state fluorescence yield (*F*_s_) and the maximum fluorescence yield (*F*_m_’) under a saturating pulse. These measurements were immediately followed by exposure to weak far-red light to determine the minimum fluorescence yield (*F*_o_’) of a high light-acclimated sample (Genty *et al.*, 1989). Following exposure to high light, leaves were dark adapted for 55–60min before measuring the maximum fluorescence yield after dark relaxation following a high light photoinhibitory treatment (*F*_vPI_/*F*_mPI_). Pre-dawn measurements were conducted immediately before the lights came on the following morning to determine *F*_v_/*F*_m_. This series of fluorescence measurements was made on day 0 before chilling, days 1, 2, 4, and 6 during chilling, and after re-establishing warm temperatures on day 7. Pre-dawn measurements were also taken the morning before the experiment (*F*_vM_/*F*_mM_) to determine *F*_v_/*F*_m_ prior to high light exposure. The quantum yield of photoinactivated PSII reaction centres (Φ_NF_) and the quantum yield of short-term dark reversible photoprotective processes (Φ_REG_) were calculated according to Kornyeyev and Holaday (2008). The proportion of open PSII reaction centres under a ‘lake’ model of interconnected PSII reaction centers (qL) was calculated according to Kramer *et al.* (2004) (see Supplementary Table S1 available at *JXB* online for calculation details).

### Field study

A field survey to evaluate *A* and pre-dawn *F*_v_/*F*_m_ in *M.×giganteus* and the other *Miscanthus* hybrids was conducted during May of 2010 using plants from a pre-existing agronomy trial. Energycane and sugarcane were not part of the field trial, as they cannot survive the local winter. The field site was a single block with one plot per genotype arranged in a rectangular grid with a row of border plants to minimize edge effects. Each genotype plot had 24 plants (6×4) and was established by Mendel Biotechnology, Inc. near Elora, Ontario, Canada in the spring of 2007. Between 4 May and 19 May, a cold front induced chilling conditions and frost occurred on 9–10 May (minimum air temperature was –1.8 °C). After 10 May, sustained chilling temperatures (<13 °C daily average) lasted until 19 May. Air temperature was recorded at a weather station 900 m away from the field site. Air temperature and relative humidity were measured inside a Stevenson screen 1.5 m above the ground, and wind speed was measured 10 m above the ground (http://climate.weatheroffice.gc.ca/climateData/dailydata_e.html?timeframe=2&Prov=ONT&StationID=41983&dlyRange=2003-10-01&Month=5&Year=2010&cmdB1=Go).

Before sunrise (05:45h) pre-dawn *F*_v_/*F*_m_ measurements were obtained on the youngest fully expanded leaves of 10 randomly chosen plants of each genotype using the PAM-2100 fluorometer with the portable leaf clip. Five randomly selected plants per genotype were chosen for gas exchange for each survey date. Gas exchange measurements began at 09:00h and were finished by 13:00h, with the measured order of genotypes alternating on every measurement date. The LI-6400 measured *A* on the youngest fully expanded leaf at 1800 μmol photons m^–2^ s^–1^ at ambient CO_2_ conditions of 380 μmol mol^–1^. The leaf to air vapour pressure difference was maintained between 0.6 kPa and 2.5 kPa, and leaf temperature was ±5 °C of the peak daytime temperature.

### Experimental design and data analysis

The growth chamber experiment consisted of five replicated trials, with one plant of each genotype per trial. Different cohorts of plants were used for each trial. Over the five trials, it was possible to measure 3–5 plants of each genotype; not all genotypes were measured in each trial due to time constraints. For the field survey, five randomly selected plants of a given genotype were measured within each respective plot. For each photosynthetic parameter measured on each day of the chilling experiment and field survey date, normality was assessed with P–P plots and Shapiro–Wilk tests at *P*≥0.01. Homogeneity of variance was assessed with a Levene’s test at *P*≥0.05. Data that met these criteria were subsequently evaluated with one-way analyses of variance (ANOVA) and the Holm–Sidak post-hoc test to assess genotypic differences. Two-way ANOVAs that met the normality and homogeneity criteria were also performed on *A* to assess injury and acclimation to chilling conditions for the growth chamber chilling experiment. For the two-way ANOVAs, both day and genotype were factors for days 0 and 7 (injury, both measured at 25 °C) and days 1 and 6 (acclimation, both measured at 11 °C). Results of ANOVAs for data that failed tests of normality and homogeneity of variance were not reported.

To evaluate the impact of chilling, the relative change (RC) from day 0 to day 6 was calculated for the parameters (*P*): *A*, CE, Φ_NF_, Φ_REG_, and qL in the growth chamber chilling experiment as follows:

$${\text{RC}}_{\text{6}}=\left[\left(P_{\text{day6}}–P_{\text{day}0}\right)/P_{\text{day}0}\right]\times \text{1}00$$ (1)

To evaluate the recovery from chilling, the RC for each above *P* was also calculated from day 0 to day 7:

$${\text{RC}}_{\text{7}}=\left[\left(P_{\text{day7}}–P_{\text{day}0}\right)/P_{\text{day}0}\right]\times \text{1}00$$ (2)

RC values were calculated from individual rounds of replication where parameters from days 0 and 6 and days 0 and 7 were obtained. As for the other photosynthetic parameters, one-way ANOVAs were performed on RC data sets that met the normality and homogeneity of variance criteria. Because *M.×giganteus* is the research standard among *Miscanthus* hybrids, simple contrasts comparing *M.×giganteus* with the other genotypes were also performed on the RC values. These are specific tests comparing the mean RC of *M.×giganteus* with the other genotypes, and were performed in addition to Holm–Sidak post-hoc tests following one-way ANOVAs. All statistical analyses were performed with SPSS Statistics version 20 (http://www-01.ibm.com/software/analytics/spss/).

## Results

### Growth chamber chilling experiment

Before chilling commenced, most genotypes had similar *A* values of 35–37 μmol m^–2^ s^–1^, with the exception of *M118* which was significantly lower than every genotype except *M115* (Table 1). On the first day of chilling, the genotypes exhibited a 32–70% reduction in *A* measured at 11 **°**C relative to 25 **°**C on day 0. Energycane had the least reduction (52%) while *M115* had the greatest (70%). Over the subsequent 5 d, *A* gradually declined in all genotypes, such that at a measurement temperature of 11 **°**C on day 6 relative to the 25 **°**C measurement on day 0, *A* was reduced 64–70% in the least affected genotypes (*M.×giganteus*, *M116*, *M147*, and energycane) but >76% in the most affected genotypes (*M115*, *M118*, and ULSC) (Table 2). *Miscanthus×giganteus* had the greatest *A* after re-establishing warm temperatures on day 7, and this was significantly greater than *M115* and *M118* (Table 2). With the exception of energycane, a higher *A* under chilling conditions corresponded to greater recovery of *A* after re-establishing warm temperatures (Tables 1 and 2). The two-way ANOVA for injury of *A* (days 0 and 7) failed Levene’s test; however, the two-way ANOVA for acclimation (days 1 and 6) met the normality and homogeneity criteria. Both day [*F*(1,37)=30.12, *P*<0.001] and genotype [*F*(6,37)=6.86, *P*<0.001] were highly significant; however, no interaction was detected [*F*(6,37)=0.27, *P*=0.95].

**Table 1.** Results of the growth chamber chilling experiment showing average values of net CO_2_ assimilation rate and carboxylation efficiency Values are mean averages ±SE with *n*=3–5, except for carboxylation efficiency on day 7 where *n*=1 or 2.

Genotype (ploidy)	Day 0 (25/20 °C)	Day 1 (12/5 °C)	Day 2 (12/5 °C)	Day 4 (12/5 °C)	Day 6 (12/5 °C)	Day 7 (25/20 °C)
	**Net CO_2_ assimilation rate, μmol m^–2^ s^–1^**					
*M×G* (3*x*)	36.6±0.4	14.7±0.8	14.1±0.2 a,b	12.3±0.3 a	12.4±1.7	30.5±3.1 a
*M116* (3*x*)	35.1±0.4	14.5±0.1	10.6±1.8 b	10.4±1.6 a,b	10.4±1.0	27.4±0.4 a,b
*M147* (2*x*)	35.3±0.8	14.3±0.9	13.2±0.7 a,b	11.4±0.5 a,b	10.8±0.6	23.8±1.4 a,b
*M115* (2*x*)	31.8±0.6	9.6±1.2	8.9±0.8 b	6.7±0.6 b	6.5±0.5	18.4±1.8 b
*M118* (4*x*)	28.1±1.6	11.6±1.2	9.6±0.9 b	7.7±1.2 a,b	6.5±1.4	17.6±1.9 b
Energycane	34.9±1.3	16.7±0.6	16.6±1.0 a	12.2±0.7 a	12.4±1.1	21.4±1.2 a,b
ULSC	35.3±2.3	12.5±1.8	9.8±1.7 b	8.9±1.5 a,b	8.5±2.3	20.5±3.9 a,b
	**Carboxylation efficiency, Δ*A* Δ*C*_i_^–1^**					
*M×G* (3*x*)	0.33±0.02 a,b,c	0.20±0.03	0.18±0.03 a,b	0.16±0.03 a	0.16±0.03	0.23±0.00
*M116* (3*x*)	0.37±0.04 a,b,c	0.25±0.02	0.15±0.06 a,b	0.16±0.02 a	0.16±0.03	0.23
*M147* (2*x*)	0.33±0.02 a,b,c	0.19±0.02	0.21±0.03 a,b	0.16±0.01 a	0.14±0.02	0.21±0.04
*M115* (2*x*)	0.30±0.00 b,c	0.13±0.01	0.11±0.03 b	0.10±0.02 a,b	0.07±0.02	0.18±0.02
*M118* (4*x*)	0.28±0.02 c	0.15±0.01	0.13±0.01 a,b	0.07±0.01 b	0.07±0.01	0.16±0.02
Energycane	0.44±0.04 a	0.27±0.02	0.23±0.02 a	0.17±0.01 a	0.15±0.04	0.17
ULSC	0.42±0.03 a,b	0.19±0.03	0.10±0.02 b	0.11±0.01 a,b	0.12±0.02	0.26±0.03

Different letters indicate significant differences between genotypes at *P*<0.05 using Holm–Sidak post-hoc tests following one-way ANOVAs that showed genotype as significant (*P*<0.05).

Measurement temperatures were 25 °C before and after chilling (day 0 and 7) and 11 °C during chilling (days 1–6).

Column heading temperatures indicate day/night leaf temperatures.

**Table 2.** The average percentage relative change (RC) in net CO_2_ assimilation rate (A) and carboxylation efficiency (CE) for each genotype in the growth chamber chilling experimentValues are mean averages ±SE with *n*= 3–5, except for CE RC_7_ where *n*= 1–2.

Genotype (ploidy)	RC_6_	RC_7_	RC_6_	RC_7_
	Net CO_2_ assimilation rate		Carboxylation efficiency	
*M×G* (3*x*)	–66.0±4.2	–16.8±6.5	–52.3±6.4	–26.3±7.2
*M116* (3*x*)	–70.4±4.2	–21.9±6.5	–62.1±7.8	–39.4±10.2
*M147* (2*x*)	–69.6±3.7	–32.8±5.1	–56.0±5.5	–38.8±7.2
*M115* (2*x*)	–79.6±4.2*	–42.0±5.7**	–75.3±6.4*	–37.9±7.2
*M118* (4*x*)	–78.0±3.7*	–37.8±5.1*	–74.7±5.5*	–47.6±7.2
Energycane	–68.5±5.2	–37.3±5.7*	–68.5±7.8	ND
ULSC	–76.3±3.7	–42.1±5.7**	–73.7±6.4*	–43.4±7.2

See the Materials and methods for calculation of RC_6_ and RC_7_.

Asterisks indicate significant differences from *M.×giganteus* using simple contrasts, *P<0.05; **P<0.01.

ND, not determined (due to equipment failure).

**Fig. 1.**
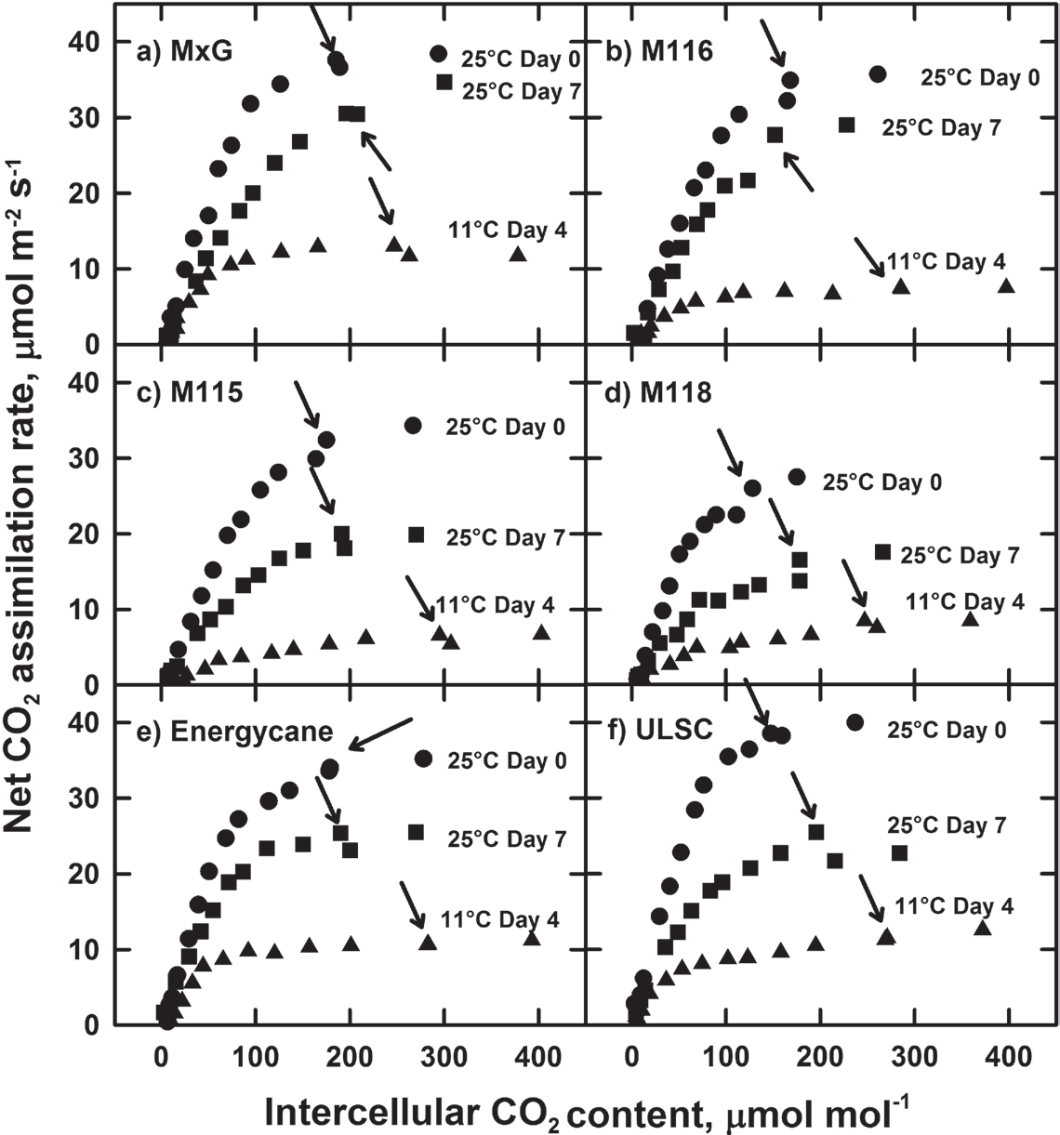
Representative responses of net CO_2_ assimilation rate to intercellular CO_2_ content (*A*/*C*_i_) in plants from the growth chamber chilling experiment. Circles, day 0 (25 °C); triangles, day 4 (11 °C); squares, day 7 (25 °C). *M147* is not shown as it has a similar response to *M116*. Curves were chosen to represent the median response of each genotype on that day and are not necessarily from the same leaf/cohort of replication. Arrows indicate the operational *C*_i_, which is the *C*_i_ corresponding to ambient CO_2_ concentrations of 380 μmol mol^–1^.

The CO_2_-saturated *A* (*A*_sat_) was 65–79% lower in plants exposed to chilling for 4 d at 11 °C, relative to their *A*_sat_ values at 25 °C the day before chilling (Fig. 1A–F). Upon return to 25 **°**C on day 7, *A*_sat_ remained depressed relative to day 0 measurements, particularly in the chilling-sensitive genotypes, *M115*, *M118*, ULSC, and energycane (Table 1). In *M115*, *M118*, and ULSC on day 4 of chilling, *A* at 11 **°**C was not CO_2_-saturated below a *C*_i_ of 300 μmol mol^–1^, in contrast to *M.×giganteus*, *M116*, and energycane, where the CO_2_ saturation point of *A* was <120 μmol mol^–1^ (Fig. 1A–F). The CE was reduced >70% by 6 d of chilling in *M115*, *M118*, and ULSC, but <60% in the triploid hybrids *M.×giganteus* and *M116* (Fig. 1A–D, F). For the most chilling-tolerant genotypes (*M.×giganteus*, *M116*, and *M147*), the relative declines in CE between day 0 and 6 were 8–16% less than the relative declines in *A*, in contrast to the chilling-sensitive genotypes where the change in CE was within 4.3% of the change in *A* on day 6 (Tables 1 and 2). When measured at 25 °C on day 7 of the experiment, *M.×giganteus* showed the smallest relative decline in CE relative to the original values at 25 °C on day 0, consistent with its superior ability to recover *A* upon rewarming (Table 2).

Relative to other genotypes, *M×giganteus* had up to 50% lower values of Φ_NF_, 4–28% higher values of Φ_REG_, and 46–69% higher values of qL on day 6 of chilling (Supplementary Table S2 available at *JXB* online). All of the chilling-tolerant genotypes (*M.×giganteus*, *M116*, and *M147*) increased Φ_REG_ between day 1 and 6 of chilling. *Miscanthus×giganteus* exhibited the largest relative increase of Φ_REG_ on day 6; its increase was >48% greater than all other genotypes (Table 3). The relative increase in Φ_NF_ from day 0 to day 6 is the smallest in triploid *M.×giganteus* and *M116*, greater in diploid *M147*, *M115*, and ULSC, and greatest in energycane and tetraploid *M118* (Table 3). The relative increase in Φ_NF_ persists most in *M118*, energycane, and ULSC after re-establishing warm temperatures on day 7 (Table 3). Upon return to warm temperatures on day 7, qL in *M.×giganteus* was 32–64% higher than in all other genotypes, with *M118* having the lowest value (Supplementary Table S2 available at *JXB* online). The greater qL of *M.×giganteus* throughout the experiment can be attributed to its greater Φ_P_ throughout the experiment, significantly higher than that of *M115* and *M118* on day 6 of chilling (Supplementary Table S3 available at *JXB* online). *M118* showed the largest decline in qL on day 6 of chilling despite an increase in Φ_REG_ on day 6 of chilling that is comparable with that of the chilling-tolerant genotypes (Table 3).

**Table 3.** The average percentage relative change (RC) in the quantum yield of NPQ associated with photoinactivated PSII under high light (Φ_NF_), the quantum yield of dark-reversible NPQ (Φ_REG_), and the proportion of open PSII reaction centres or photochemical quenching under a ‘lake’ model of connected antenna systems (qL) for each genotype in the growth chamber chilling experimentValues are means ±SE with *n*=2–5.

Genotype(ploidy)	RC_6_	RC_7_	RC_6_	RC_7_	RC_6_	RC_7_
	Φ_NF_		Φ_REG_		qL	
*M×G* (3*x*)	263.8±255.4	68.7±133.4	6.4±2.2	8.4±0.4	–39.1±15.5	12.8±25.8
*M116* (3*x*)	217.6±312.8	76.7±163.4	3.3±1.7	12.4±0.5	–63.1±18.9	–40.1±31.6
*M147* (2*x*)	285.2±197.8	179.9±103.3	2.1±2.3	9.6±2.7	–56.4±12.0	–11.5±20.0
*M*115 (2*x*)	427.0±221.2	188.3±115.5	–5.3±3.1*	9.1±3.2	–64.3±13.4	–13.0±22.3
*M118* (4*x*)	729.6±197.8	329.7±103.3	3.3±1.4	12.9±2.8	–70.9±12.0	–7.4±20.0
Energycane	755.2±221.2	325.6±115.5	2.6±4.5	14.0±4.6	–20.8±12.0	29.9±20.0
ULSC	621.3±197.8	344.0±103.3	–3.5±1.9*	7.0±2.2	–41.7±13.4	26.6±22.3

See the Materials and methods for calculation of RC_6_ and RC_7_.

Asterisks indicate significant differences from *Miscanthus×giganteus* using simple contrasts, *P <0.05.

Pre-dawn *F*_v_/*F*_m_ values measured at 20 °C were similar (0.82–0.83 on day 0 and 0.79–0.81 on day 7) in all genotypes, but were 7–10% lower in *M115* on days 4 and 6 compared with *M.×giganteus* (Supplementary Table S2 available at *JXB* online). Fluorescence yields after 55–60min of dark adaptation (*F*_vPI_/*F*_mPI_) ranged from 0.77 to 0.80 on day 0 and from 0.66 to 0.72 on day 7, and were 9–22% lower in *M115* on days 2–6 compared with *M.×giganteus* (Supplementary Table S2 available at *JXB* online).

### Field study

**Fig. 2.**
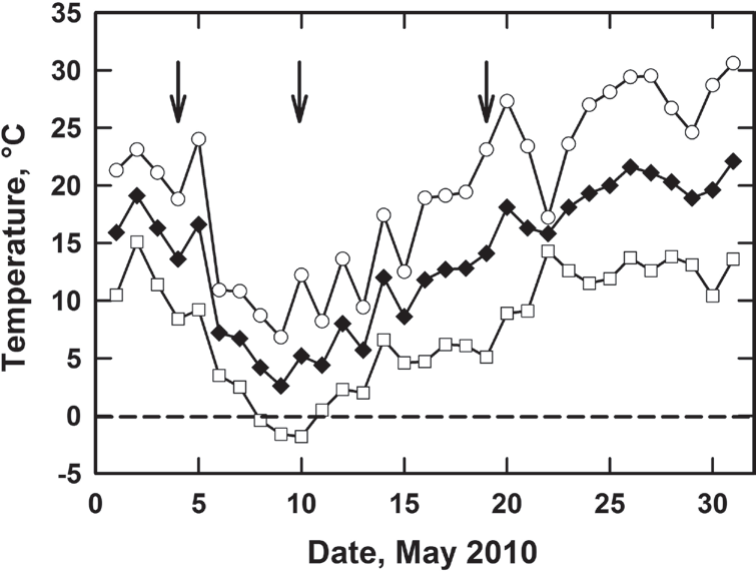
Air temperature parameters at the Elora, Ontario field site from May 2010. Open circles indicate daily highs, open squares daily lows, and solid diamonds indicate daily mean averages. Data from Environment Canada National Climate Data and Information Archive: (http://climate.weatheroffice.gc.ca/climateData/dailydata_e.html?timeframe=2&Prov=ONT&StationID=41983&dlyRange=2003-10-01&Month=5&Year=2010&cmdB1=Go).

New shoots of the *Miscanthus* genotypes emerged just prior to 1 May 2010 at the Elora field site during a spring warm front (Fig. 2). On 4 May, *M.×giganteus* and *M116* had the highest *A*, which was significantly higher than that of *M118* at leaf temperatures of 20.1±2.4 **°**C (Table 4). Temperatures declined in the days following 4 May as a cold front moved across Ontario, such that frost occurred on the night of 9–10 May (Fig. 2). The coldest point of the night occurred at 01:00h on 10 May (–1.8 °C) and was associated with a wind speed of 4 km h^–1^ and a relative humidity of 87%. Shoots were visibly damaged on the morning of 10 May and all genotypes except an *M. sinensis* (*M. sin15*) lacked photosynthesis and were respiring at measurement temperatures of 12.8±1 **°**C (Table 4). Conditions warmed the week after the frost event, and a new set of leaves grew to replace those killed by the frost of 9–10 May (Fig. 2). On 19 May, *M.×giganteus* exhibited the greatest *A* values in newly produced leaves as *M147*, *M115*, and *M118* exhibited *A* values that were significantly lower, less than half that of *M.×giganteus* at leaf temperatures of 24.4±1.3 **°**C (Table 4). Pre-dawn *F*_v_/*F*_m_ yields were similar between genotypes on 4 May and within 20% of maximum values for C_4_ plants (Table 4). On the morning of 10 May, *F*_v_/*F*_m_ values in all genotypes were depressed to ≤0.21 by the overnight frost (Table 4). Values of pre-dawn *F*_v_/*F*_m_ in the new growth on 19 May remained below values on 4 May and were highest in the triploids *M.×giganteus* and *M116*, significantly higher than in the diploid *M115* (Table 4).

**Table 4.** Net CO_2_ assimilation rate (A) and pre-dawn F_v_/F_m_ in field-grown plants before, during, and after a frost event in May 2010 (mean ±SE, n=5 for A and 10 for F_v_/F_m_)

Genotype (ploidy)	4 May	10 May	19 May
	**Net CO_2_ assimilation rate, *A*, μmol m^–2^ s^–1^**		
*M×G* (3*x*)	19.6±1.4 a	–0.5±0.06	13.8±1.4 a
*M*116 (3*x*)	19.2±0.3 a	–0.2±0.03	6.6±2.0 a,b
*M147* (2*x*)	15.0±1.3 a,b	0.0±0.08	2.4±1.0 b
*M115* (2*x*)	14.6±1.3 a,b	–0.2±0.09	4.5±1.3 b
*M118* (4*x*)	12.6±1.4 b	–0.3±0.05	4.6±2.3 b
*M. sin15*	13.1±1.6 b	0.7±0.52	9.8±1.2 a,b
	**Pre-dawn *F*_v_*/F*_m_, relative units**		
*MxG* (3*x*)	0.67±0.02 a,b	0.18±0.03	0.46±0.04 a
*M116* (3*x*)	0.65±0.02 a,b	0.16±0.03	0.43±0.04 a
*M147* (2*x*)	0.64±0.02 a,b	0.11±0.02	0.31±0.04 a,b
*M*115 (2*x*)	0.63±0.02 a,b	0.16±0.02	0.23±0.05 b
*M118* (4*x*)	0.68±0.01 a	0.21±0.03	0.36±0.06 a,b
*M. sin15*	0.61±0.01 b	0.16±0.02	0.23±0.04 b

Different letters indicate significant differences between genotypes at *P*<0.05 using Holm–Sidak post-hoc tests following one-way ANOVAs that showed genotype as significant (*P*<0.05).

Leaf temperatures averaged 20.1±2.4 **°**C on 4 May, 12.9±1.2 **°**C on 10 May, and 24.5±1.4 **°**C on 19 May.

Pre-dawn *F*_v_/*F*_m_ measurement temperatures were 9.6 °C on 4 May, –0.5 °C on 10 May, and 7.9 °C on 19 May.

## Discussion

Of the genotypes in this study, the triploid hybrids *M.×giganteus* and *M116* are most chilling tolerant, as indicated by their ability to maintain higher *A* during chilling and recover *A* after exposure to chilling and frost. *M115* and *M118* are the most chilling-sensitive *Miscanthus* hybrids in the growth chamber chilling experiment, and the field survey is consistent with this. Only *M147* shows some discrepancy in cold sensitivity between the growth chamber experiment and the field survey. It was one of the least affected in the growth chamber but the most chilling sensitive in the field survey. Energycane performed similarly to *M.×giganteus* under chilling but could not recover *A* to a similar extent 1 d after re-establishing the warm growth temperatures. Upland sugarcane was also chilling sensitive and showed a similar response to *M115* and *M118*.

*Miscanthus sinensis* and *M. sacchariflorus* are part of the core group of *Miscanthus* species that have a wide geographic range in east Asia (Hodkinson *et al.*, 2002*a*; Clifton-Brown *et al.*, 2013). *Miscanthus×giganteus* and *M116* (‘Nagara’) are both triploid hybrids of a tetraploid Japanese *M. sacchariflorus* and a diploid *M. sinensis* (Hodkinson *et al.*, 2002*b*; Rayburn *et al.*, 2009; G. van Koeverden, personal communication, August 2010; Nishiwaki *et al.*, 2011; unpublished flow cytometry data). The more chilling-sensitive Amuri lines *M115* and *M147* are diploids from *M. sacchariflorus* mothers collected from the Amur River basin of eastern Siberia at 50 °N but with unknown *M. sinensis* fathers (G. van Koeverden, personal communication, August 2010). Tetraploid *M118* also exhibited greater chilling sensitivity than the triploids. This genotype has the same maternal *M. sacchariflorus* parent as *M116* (‘Nagara’) but probably a different *M. sinensis* father (G. van Koeverden, personal communication, August 2010). Purdy *et al.* (2013) also observed superior tolerance of short-term chilling in *M.×giganteus* compared with a diploid *M. sinensis*, tetraploid *M. sacchariflorus*, and a triploid intraspecific *M. sinensis* hybrid. In their study, *M.×giganteus* had a greater leaf extension rate and recovered *A* best after 1 d at 12 °C (Purdy *et al.*, 2013). Why the triploid *M. sacchariflorus×M. sinensis* hybrids exhibit greater chilling tolerance is uncertain. The location of origin of the *M. sinensis* fathers is unknown, such that the contribution of each parent species to chilling tolerance cannot be evaluated. Whatever the reason, the results indicate that something is unique to the triploid state that enhances chilling tolerance in the triploid hybrids studied here.

Although ULSC shows a depression of *A* after 2 d of chilling that is comparable with other subtropical sugarcane varieties, it is 17% more depressed than energycane and this may represent the upper limit on chilling tolerance for commercial sugarcane (*S. officinarum*). After 2 d of chilling warm-grown (30 °C) sugarcane to 10 °C, *A* decreased by 90% in a tropical variety of *S. officinarum* compared with 70% in a tropical–subtropical hybrid of *S. officinarum×S. spontaneum×S. barberi*, and 75% in a subtropical variety of *S. sinense* when measured at 10 °C (Du *et al.*, 1999*a*). This compares with 71% in ULSC and 54% in energycane when measured at 11 °C. In the field, sustained depression of *A* was observed in cv. H67-5630 (*S. officinarum*) of commercial sugarcane from Hawaii following cold nights that were only ~5 °C lower than the daytime high, which was 14 °C in the winter and 21 °C in the summer (Grantz, 1989). Young sugarcane plantlets (*S. officinarum*) (<6 months of age) had a ~33% lower qP (equivalent to qL, assuming a ‘puddle’ model; Kramer *et al.*, 2004) at moderate light intensities (400 μmol m^–2^ s^–1^) when grown at 15 °C compared with 27 °C (Ebrahim *et al.*, 1998). Given the acute chilling sensitivity apparent in *S. officinarum*, it is concluded that complex hybrids of *S. officinarum* with *S. sinense*, *S. barberi*, and *S. spontaneum* are more chilling tolerant among *Saccharum* crops.

Among energycane cultivars, the ‘Ho 02’ series of F_1_ hybrids appear to be most cold tolerant and most productive (Khan *et al.*, 2013; Knoll *et al.*, 2013). ‘Ho 02-144’, a full sibling of ‘Ho 02-113’ used for this study, was much more tolerant of 1 week at 0 °C than ‘L79-1002’, another complex *S. officinarum×S. spontaneum×S. barberi×S. sinense* F_1_ hybrid of commercial sugarcane and *S. spontaneum* (Bischoff *et al.*, 2008; Khan *et al.*, 2013). Whereas ‘L79-1002’ became chlorotic and died, ‘Ho 02-144’ survived and expressed a unique suite of cold-responsive genes (Khan *et al.*, 2013). This cold tolerance may be due to the Himalayan origins of the *S. spontaneum* parent for the ‘Ho 02’ varieties and the likely more tropical Taiwanese origins of the *S. spontaneum* parent for ‘L79-1002’ (Bischoff *et al.*, 2008; Hale *et al.*, 2013). For the ‘Ho 02’ varieties, the maternal parent was the Himalayan *S. spontaneum*, whereas for ‘L79-1002’ the Taiwanese *S. spontaneum* was the paternal parent. Although further work is needed to understand how chilling tolerance combines in *Saccharum* hybrids, it appears that continued breeding of hybrids with *S. spontaneum* mothers from cooler climates may allow energycanes to be even more productive in cooler temperate climates.

In all genotypes except energycane, the decline in *A* and CE follow a similar pattern during chilling and this may reflect similar mechanisms of cold lability. The CE in C_4_ plants is modelled to reflect the *in vivo* activity of PEPCase and is normally insensitive to short-term deviations in temperature because PEPCase operates below its *K*_m_ at atmospheric CO_2_ levels (Laisk and Edwards, 1997; von Caemmerer, 2000). The extractable activity of PEPCase does not greatly decline in cold-sensitive sugarcane chilled to 10/10 °C or in *E. crus-galli* chilled to 7 °C (Potvin *et al.*, 1986; Du *et al.*, 1999*a*). In both *M.×giganteus* and cold-sensitive maize grown at 14/11 °C, RNA levels and protein content of PEPCase do not greatly decline relative to plants grown at 25/20 °C (Naidu *et al.*, 2003). Given the lack of evidence for a reduction in PEPCase content, it is hypothesized that the decline in CE results from a reduction in PEP regeneration, which will also slow PEPCase activity. PEP regeneration greatly reflects PPDK activity (von Caemmerer, 2000).

The capacity of PPDK to regenerate PEP may not only be responsible for maintaining CE under chilling, but may also co-limit *A*_sat_, possibly in combination with electron transport capacity. Increases in protein content and extractable activity of PPDK correspond to the maintenance and recovery of *A*_sat_ in *M.×giganteus* grown at or briefly chilled to 14/11 °C (Naidu *et al.*, 2003; Wang *et al.*, 2008). The present data support this pattern; by day 6 the decline in CE in the chilling-sensitive genotypes follows the decline in *A*_sat_. Whereas *M.×giganteus* appears to maintain both active PPDK and an intact thylakoid apparatus as indicated by sustained Φ_REG_ and qL (this study), the chilling-sensitive genotypes show greater increases in Φ_NF_ and declines in Φ_REG_ and/or qL, indicating sensitivity of the thylakoid apparatus and a possible lesion in electron transport capacity. A lesion in electron transport could limit PEP regeneration and RuBP regeneration, both of which have been hypothesized to control *A*_sat_ in C_4_ plants at high light (von Caemmerer, 2000; Sage and Kubien, 2007; Sage *et al.*, 2011).

A reduction in the maximum fluorescence yield after 1h in the dark (*F*_vPI_/*F*_mPI_) demonstrates sustained NPQ after high energy quenching (qE) and state transition quenching (qT) has relaxed (Maxwell and Johnson, 2000; Müller *et al.*, 2001; Takahashi and Murata, 2008). Increases in photoinactivation (Φ_NF_) are associated with declines in *F*_vPI_/*F*_mPI_ over the chilling period; when this occurs with declining *F*_v_/*F*_m_, it suggests a greater lesion in the thylakoid apparatus, which may be due to cumulative photodamage from oxidative stress. Increases in Φ_NF_ arise at the expense of Φ_REG_, such that maintaining *F*_vPI_/*F*_mPI_ maintains Φ_REG_ and suggests a greater capacity for photoprotective processes such as xanthophyll cycling and the associated physical changes to PSII to dissipate excess light energy safely (Long *et al.*, 1994; Huner *et al.*, 1998; Holt *et al.*, 2005; Horton and Ruban, 2005; Demmig-Adams *et al.*, 2006; Takahashi and Badger, 2011).

Across all genotypes, the fluorescence parameter that best corresponds to chilling sensitivity is the value of Φ_NF_ on day 6 of chilling. The increase in Φ_NF_ and decline in Φ_REG_ during chilling in *M115* and ULSC indicates that photoprotective processes such as xanthophyll cycling and/or reaction centre quenching fail to dissipate excess light energy sufficiently and photoprotect PSII from photoinactivation. This cumulative photoinactivation results in photodamage in *M115* as *F*_v_/*F*_m_ declines toward the end of chilling. The increase in Φ_REG_ in *M.×giganteus* under chilling is corroborated by previous work showing a 20-fold increase in zeaxanthin when grown at 10/8 °C compared with 14/11 °C or 25/20 °C (Farage *et al.*, 2006). A greater capacity for Φ_REG_ combined with higher *F*_v_/*F*_m_ in the field indicate that *M.×giganteus* and probably *M116* have strong but flexible xanthophyll cycles to prevent photodamage, ultimately achieving higher pre-dawn *F*_v_/*F*_m_ yields and midday *A* after sustained chilling (Demmig-Adams *et al.*, 2012).

To perform well in cold climates, *Miscanthus* plants need to be frost tolerant as well as chilling tolerant; however, the mechanisms of chilling and frost tolerance differ, and thus could segregate differentially among genotypes. Chilling tolerance is associated with safely dissipating excess light energy to prevent oxidative damage while maintaining active photosynthetic enzymes and fluid membranes to sustain electron transport (Ensminger *et al.*, 2006). Frost tolerance is associated with either controlling or preventing extracellular ice formation to mitigate damage to proteins and lysing of membranes (Ruelland *et al.*, 2009). *Miscanthus sinensis* has been reported to have a leaf frost LT_50_ of –9.3 **°**C, some 2 °C lower than that of *M. sacchariflorus* and 1.5 °C lower than that of *M.×giganteus* (Farrell *et al.*, 2006). Greater frost tolerance of *M. sinensis* over *M.×giganteus* has also been reported by Zub *et al.* (2012). Although leaves of *Miscanthus* have been reported to survive temperatures of –6 °C to –9 **°**C in controlled environments (Farrell *et al.*, 2006, Zub *et al.*, 2012), leaves of all the field-grown *Miscanthus* hybrids of the current study except one variety of *M. sinensis* (*M. sin15*) were killed by high light and chilling temperatures following an overnight frost event with an air temperature of –1.8 **°**C in the field. Because of the still early morning air, leaf temperatures in the field were probably –4 °C to –8 **°**C from radiation loss to a cold sky and limited convective heat transfer with the surrounding air. Leaf temperatures below the dew point probably fostered leaf ice crystal formation, and under these conditions frost tolerance may be slightly less than previously reported (Farrell *et al.*, 2006, Zub *et al.*, 2012), and not correlated with patterns of chilling tolerance.

Overall cold tolerance in *Miscanthus* is the combination of chilling tolerance, frost tolerance, and the ability for rhizomes to overwinter successfully. Here the chilling tolerance of five *Miscanthus* hybrids, energycane, and upland sugarcane was examined, and triploid *Miscanthus* hybrids were found to be most chilling tolerant. Future work should target how the traits required for full spectrum cold tolerance combine among *M. sinensis*×*M. sacchariflorus* hybrids. Based on chilling tolerance alone, *M.×giganteus* was found to be the better cultivar of those studied for planting in climates that experience severe chilling events. Breeding efforts to improve overall cold tolerance should aim to generate new triploid hybrids after *M.×giganteus* but with superior leaf frost and rhizome freezing tolerance (Clifton-Brown and Lewandowski, 2000).

## Supplementary data

Supplementary data are available at *JXB* online.

Table S1. Background, tables, and equations used for calculation of chlorophyll fluorescence parameters in the growth chamber chilling experiment.

Table S2. Average fluorescence parameters qL, Φ_REG_, and Φ_NF_ for each day of the growth chamber chilling experiment.

Table S3. Average fluorescence parameters used for calculation of qL, Φ_REG_, and Φ_NF_ for each day of the growth chamber chilling experiment.

Supplementary Data
