# Supplementary material for: Chilling and frost tolerance in Miscanthus and Saccharum genotypes bred for cool temperate climates
Source: J Exp Bot. 2014 Mar 18;65(13):3749–58. doi: 10.1093/jxb/eru105 (PMC4085960; doi:10.1093/jxb/eru105)
Supplement: Supplementary Data [file supp_65_13_3749__index.html]

Chilling and frost tolerance in Miscanthus and Saccharum genotypes bred for cool temperate climates — Chilling and frost tolerance in Miscanthus and Saccharum genotypes bred for cool temperate climates — Supplementary Data 

# Chilling and frost tolerance in *Miscanthus* and *Saccharum* genotypes bred for cool temperate climates

## Supplementary Data

Data files

**Files in this Data Supplement:**

- Supplementary Data - Supplementary Data
