# Supplementary material for: Chilling and frost tolerance in Miscanthus and Saccharum genotypes bred for cool temperate climates
Source: J Exp Bot. 2014 Mar 18;65(13):3749–58. doi: 10.1093/jxb/eru105 (PMC4085960; doi:10.1093/jxb/eru105)
Supplement: Supplementary Data [file supp_eru105_jexbot117184_file001.pdf]

Supplementary Data

Table S1 Definitions of fluorescence parameters used for the growth chamber chilling experiment and field survey

Parameter	Definition
Φ_{NF}	Maximum potential quantum yield of NPQ associated with photoinactivated PSII
Φ_{REG}	Maximum potential quantum yield of NPQ associated with dark-reversible photoprotective mechanisms
qL	The proportion of open PSII reaction centers or photochemical quenching under a 'lake' model of interconnected antenna
F_{vM}/F_{mM}	Pre-experiment pre-dawn maximum quantum yield of PSII
F_{vPI}/F_{mPI}	Maximum quantum yield of PSII after photoinhibitory treatment and ~1 hour of relaxation in the dark
F_v/F_m	Pre-dawn maximum quantum yields of PSII throughout the chilling experiment or in the field survey
$F_v'/F_m' = \Phi_{PSII}$	Maximum potential quantum yield of PSII of an irradiance acclimated sample
$(F_m'-F_s)/F_m' = 1-(F_s/F_m') = \Phi_P$	Realized quantum yield of PSII of an irradiance acclimated sample

To determine any appreciable fraction of NPQ associated with sustained photoinhibition from chilling, the parameter Φ_{CI} was first calculated after Φ_{WI}, as in Busch *et al.*, (2009). Φ_{CI} was negligible (≤0.01) over the entire experiment and therefore not incorporated further. Pre-chilling F_{vM}/F_{mM} values ranged from 0.80 to 0.83 and were indistinguishable from F_v/F_m values after exposure to highlight on day 0. Pre-chilling F_{vM}/F_{mM} values were thus used as the maximum unstressed reference point for calculation of NPQ associated with photoinactivated PSII (Φ_{NF}) as suggested by Kornyeyev & Holaday, (2008). Calculation of both Φ_{NF} and Φ_{REG} did not include

F_s/F_o' (oxidized Q_A pool) to better infer mechanisms underlying this NPQ (Kornyeyev *et al.*, 2006) and was calculated after Kornyeyev and Holaday, (2008)

$$\Phi_{NF} = \left(1 - \frac{F_{vPI}/F_{mPI}}{F_{vM}/F_{mM}}\right)\left(\frac{F_v'/F_m'}{F_{vPI}/F_{mPI}}\right) \quad \text{Eq. 1}$$

$$\Phi_{REG} = \left(1 - \frac{F_v'/F_m'}{F_{vPI}/F_{mPI}}\right) \quad \text{Eq. 2}$$

qL was calculated after Kramer *et al.,* (2004)

$$qL = qP\left(\frac{F_o'}{F_s}\right) \quad \text{Eq. 3}$$

where,

$$qP = \left(F_m' - F_s\right)/\left(F_m' - F_o'\right) \quad \text{Eq. 4}$$

Table S2 Results of the growth chamber chilling experiment showing mean values of $\Phi_{NF,}$ $\Phi_{REG,}$ and qL. Values are mean averages (± SE) with N = 2-5. Different letters indicate significant differences between genotypes at $p < 0.05$ using Holm-Sidak post-hoc tests following one-way ANOVAs that showed genotype as significant ($p < 0.05$). Column heading temperatures indicate day/night leaf temperatures.

**Genotype (Ploidy)**	**Day 0 (25/20°C)**	**Day 1 (12/5°C)**	**Day 2 (12/5°C)**	**Day 4 (12/5°C)**	**Day 6 (12/5°C)**	**Day 7 (25/20°C)**
			**Φ_{NF}, relative units**			
MxG (3x)	0.04 (0.01)	0.04 (0.01)	^{a,b}0.05 (0.01)	0.08 (0.02)	^{a,b}0.10 (0.02)	0.08 (0.01)
*M*116 (3x)	0.03 (0.01)	0.04 (0.01)	^{b}0.03 (0.01)	0.07 (0.02)	^{b}0.08 (0.02)	0.06 (0.01)
*M*147 (2x)	0.02 (0.00)	0.04 (0.00)	^{a,b}0.06 (0.01)	0.10 (0.02)	^{b}0.10 (0.02)	0.05 (0.01)
*M*115 (2x)	0.03 (0.00)	0.04 (0.00)	^{a}0.09 (0.01)	0.16 (0.02)	^{a}0.20 (0.02)	0.08 (0.01)
*M*118 (4x)	0.03 (0.00)	0.04 (0.00)	^{a,b}0.06 (0.01)	0.09 (0.02)	^{a,b}0.14 (0.02)	0.07 (0.01)
Energycane	0.02 (0.00)	0.03 (0.00)	^{a,b}0.07 (0.01)	0.09 (0.02)	^{a,b}0.13 (0.02)	0.06 (0.01)
ULSC	0.02 (0.00)	0.05 (0.00)	^{a,b}0.07 (0.01)	0.17 (0.02)	^{a,b}0.14 (0.02)	0.10 (0.01)
			**Φ_{REG}, relative units**			
MxG (3x)	^{a}0.51 (0.02)	^{a,b}0.49 (0.02)	^{a}0.53 (0.02)	^{a}0.54 (0.02)	^{a}0.55 (0.02)	^{a}0.56 (0.02)
*M*116 (3x)	^{a}0.51 (0.02)	^{a}0.51 (0.02)	^{a}0.52 (0.03)	^{a}0.52 (0.02)	^{a,b}0.53 (0.02)	^{a}0.58 (0.03)
*M*147 (2x)	^{a}0.52 (0.01)	^{a}0.49 (0.02)	^{a}0.49 (0.02)	^{a}0.52 (0.01)	^{a}0.53 (0.01)	^{a}0.57 (0.02)
*M*115 (2x)	^{a}0.52 (0.02)	^{a}0.49 (0.02)	^{a}0.51 (0.02)	^{a}0.50 (0.02)	^{a,b}0.49 (0.02)	^{a}0.57 (0.02)
*M*118 (4x)	^{a}0.47 (0.01)	^{a,b}0.45 (0.02)	^{a}0.47 (0.02)	^{a}0.49 (0.01)	^{a,b}0.49 (0.01)	^{a}0.54 (0.02)
Energycane	^{b}0.40 (0.01)	^{c}0.34 (0.02)	^{b}0.36 (0.02)	^{b}0.39 (0.01)	^{c}0.40 (0.01)	^{b}0.45 (0.02)
ULSC	^{a}0.47 (0.02)	^{b,c}0.41 (0.02)	^{a}0.45 (0.02)	^{a}0.47 (0.02)	^{c,b}0.46 (0.02)	^{a,b}0.51 (0.02)
			**qL, relative units**			
MxG (3x)	0.22 (0.02)	^{a}0.13 (0.03)	0.12 (0.03)	0.10 (0.00)	^{a}0.13 (0.03)	0.25 (0.04)
*M*116 (3x)	0.19 (0.03)	^{a,b}0.10 (0.01)	0.08 (0.02)	0.04 (0.00)	^{a,b}0.06 (0.01)	0.14 (0.03)
*M*147 (2x)	0.16 (0.01)	^{b}0.06 (0.01)	0.13 (0.05)	0.05 (0.00)	^{a,b}0.07 (0.01)	0.14 (0.02)
*M*115 (2x)	0.16 (0.01)	^{b}0.06 (0.02)	0.04 (0.02)	0.05 (0.02)	^{b}0.06 (0.02)	0.14 (0.03)

*M*118 (4x)	0.15	(0.01)	[b]0.06	(0.01)	0.06	(0.01)	0.06	(0.02)	[b]0.04	(0.01)	0.14	(0.04)
Energycane	0.09	(0.01)	[b]0.05	(0.01)	0.05	(0.01)	0.06	(0.01)	[b]0.07	(0.01)	0.11	(0.01)
ULSC	0.15	(0.03)	[a,b]0.08	(0.01)	0.09	(0.01)	0.07	(0.01)	[a,b]0.07	(0.01)	0.17	(0.03)

Table S3 Results of the growth chamber chilling experiment showing mean values of F_{vPI}/F_{mPI}, F_v/F_m, Φ_{PSII}, and Φ_P. Values are mean averages (± SE) with N = 2-5. Different letters indicate significant differences between genotypes at $p < 0.05$ using Holm-Sidak post-hoc tests following one-way ANOVAs that showed genotype as significant ($p < 0.05$). Column heading temperatures indicate day/night leaf temperatures.

Genotype	Day 0 (25/20°C)		Day 1 (12/5°C)		Day 2 (12/5°C)		Day 4 (12/5°C)		Day 6 (12/5°C)		Day 7 (25/20°C)	
							**F_{vPI}/F_{mPI}, relative units**					
MxG (3x)	0.77	(0.01)	0.77	(0.00)	0.74	(0.01)	0.70	(0.01)	0.64	(0.03)	0.68	(0.05)
*M*116 (3x)	0.77	(0.00)	0.76	(0.00)	0.76	(0.01)	0.70	(0.02)	0.67	(0.03)	0.71	(0.02)
*M*147 (2x)	0.78	(0.00)	0.77	(0.01)	0.72	(0.01)	0.65	(0.02)	0.65	(0.02)	0.72	(0.02)
*M*115 (2x)	0.77	(0.01)	0.75	(0.01)	0.67	(0.03)	0.56	(0.05)	0.50	(0.05)	0.66	(0.02)
*M*118 (4x)	0.78	(0.00)	0.76	(0.00)	0.73	(0.01)	0.68	(0.01)	0.60	(0.04)	0.70	(0.01)
Energycane	0.79	(0.00)	0.77	(0.01)	0.74	(0.01)	0.60	(0.03)	0.64	(0.01)	0.67	(0.03)
ULSC	0.80	(0.00)	0.78	(0.01)	0.71	(0.02)	0.67	(0.01)	0.62	(0.02)	0.72	(0.01)
							**F_v/F_m, relative units**					
MxG (3x)	0.83	(0.00)	0.79	(0.00)	0.78	(0.01)	0.77	(0.01)	0.73	(0.01)	0.81	(0.00)
*M*116 (3x)	0.82	(0.00)	0.79	(0.01)	0.79	(0.00)	0.76	(0.01)	0.72	(0.05)	0.81	(0.01)
*M*147 (2x)	0.82	(0.00)	0.78	(0.02)	0.78	(0.00)	0.75	(0.01)	0.74	(0.01)	0.80	(0.02)
*M*115 (2x)	0.82	(0.00)	0.77	(0.01)	0.76	(0.02)	0.69	(0.02)	0.68	(0.01)	0.80	(0.00)
*M*118 (4x)	0.82	(0.00)	0.78	(0.01)	0.77	(0.01)	0.73	(0.02)	0.71	(0.02)	0.80	(0.00)
Energycane	0.83	(0.01)	0.78	(0.02)	0.78	(0.01)	0.72	(0.02)	0.70	(0.02)	0.79	(0.02)
ULSC	0.82	(0.01)	0.79	(0.01)	0.76	(0.02)	0.75	(0.01)	0.70	(0.02)	0.81	(0.00)
							**Φ_{PSII}, relative units**					
MxG (3x)	0.38	(0.01)	^{b,c}0.39	(0.02)	^{b}0.35	(0.02)	^{a,b}0.32	(0.02)	^{b,c}0.29	(0.02)	0.30	(0.02)
*M*116 (3x)	0.38	(0.02)	^{b,c}0.39	(0.02)	^{b}0.37	(0.02)	^{a,b}0.34	(0.02)	^{a,b,c}0.31	(0.02)	0.30	(0.02)
*M*147 (2x)	0.37	(0.01)	^{c}0.39	(0.01)	^{b}0.37	(0.02)	^{a,b}0.31	(0.02)	^{b,c}0.31	(0.02)	0.31	(0.02)
*M*115 (2x)	0.37	(0.01)	^{c}0.38	(0.02)	^{b}0.33	(0.02)	^{b}0.28	(0.02)	^{c}0.25	(0.02)	0.28	(0.02)

*M*118 (4x)	0.41	(0.01)	[b,c]0.42	(0.01)	[a,b]0.39	(0.02)	[a,b]0.34	(0.02)	[a,b,c]0.31	(0.02)	0.33	(0.02)
Energycane	0.42	(0.01)	[a,b]0.47	(0.02)	[a]0.40	(0.02)	[a,b]0.36	(0.02)	[a,b]0.34	(0.02)	0.36	(0.02)
ULSC	0.48	(0.01)	[a]0.50	(0.01)	[a]0.48	(0.02)	[a]0.37	(0.02)	[a]0.38	(0.02)	0.37	(0.02)
**Φ_P, relative units**												
MxG (3x)	0.12	(0.01)	0.08	(0.01)	0.06	(0.02)	0.05	(0.01)	[a]0.05	(0.01)	0.10	(0.02)
*M*116 (3x)	0.10	(0.02)	0.06	(0.01)	0.04	(0.02)	0.02	(0.01)	[a,b]0.03	(0.01)	0.06	(0.02)
*M*147 (2x)	0.09	(0.01)	0.04	(0.01)	0.07	(0.02)	0.02	(0.01)	[a,b]0.03	(0.01)	0.06	(0.01)
*M*115 (2x)	0.09	(0.01)	0.03	(0.01)	0.02	(0.02)	0.02	(0.01)	[b]0.02	(0.01)	0.05	(0.01)
*M*118 (4x)	0.10	(0.01)	0.04	(0.01)	0.03	(0.02)	0.03	(0.01)	[b]0.02	(0.01)	0.06	(0.01)
Energycane	0.10	(0.01)	0.06	(0.01)	0.06	(0.02)	0.04	(0.01)	[a,b]0.04	(0.01)	0.09	(0.01)
ULSC	0.08	(0.01)	0.05	(0.01)	0.05	(0.02)	0.04	(0.01)	[a,b]0.04	(0.01)	0.06	(0.01)

References

Busch F, Huner NPA, Ensminger I. 2009. Biochemical constrains limit the potential of the photochemical reflectance index as a predictor of effective quantum efficiency of photosynthesis during the winter spring transition in Jack pine seedlings. *Functional Plant Biology* **36**, 1016-1026.

Kornyeyev D, Holaday AS. 2008. Corrections to current approaches used to calculate energy partitioning in photosystem 2. *Photosynthetica* **46**, 170-178.

Kornyeyev D, Logan BA, Tissue DT, Allen RD, Holaday AS. 2006. Compensation for PSII photoinactivation by regulated non-photochemical dissipation influences the impact of photoinactivation on electron transport and CO2 assimilation. *Plant and Cell Physiology* **47**, 437-446.

Kramer DM, Johnson G, Kiirats O, Edwards GE. 2004. New fluorescence parameters for the determination of QA redox state and excitation energy fluxes. *Photosynthesis Research* **79**, 209-218.
